# Training‐induced improvements in knee extensor force accuracy are associated with reduced vastus lateralis motor unit firing variability

**DOI:** 10.1113/EP090367

**Published:** 2022-08-12

**Authors:** Isabel A. Ely, Eleanor J. Jones, Thomas B. Inns, Síobhra Dooley, Sarah B. J. Miller, Daniel W. Stashuk, Philip J. Atherton, Bethan E. Phillips, Mathew Piasecki

**Affiliations:** ^1^ Centre of Metabolism, Ageing and Physiology, MRC‐Versus Arthritis Centre for Musculoskeletal Ageing Research, National Institute for Health Research (NIHR) Nottingham Biomedical Research Centre School of Medicine University of Nottingham Derby UK; ^2^ School of Agriculture, Food Science and Veterinary Medicine University College Dublin Dublin Ireland; ^3^ Department of Systems Design Engineering University of Waterloo Waterloo Canada

**Keywords:** electromyography, firing rate variability, motor unit, muscle force accuracy, neuromuscular function

## Abstract

**New Findings:**

- **What is the central question of this study?**
  Can bilateral knee extensor force accuracy be improved following 4 weeks of unilateral force accuracy training and are there any subsequent alterations to central and/or peripheral motor unit features?
- **What is the main finding and its importance?**
  In the trained limb only, knee extensor force tracking accuracy improved with reduced motor unit firing rate variability in the vastus lateralis, and there was no change to neuromuscular junction transmission instability. Interventional strategies to improve force accuracy may be directed to older/clinical populations where such improvements may aid performance of daily living activities.

**Abstract:**

Muscle force output during sustained submaximal isometric contractions fluctuates around an average value and is partly influenced by variation in motor unit (MU) firing rates. MU firing rate (FR) variability seemingly reduces following exercise training interventions; however, much less is known with respect to peripheral MU properties. We therefore investigated whether targeted force accuracy training could lead to improved muscle functional capacity and control, in addition to determining any alterations of individual MU features. Ten healthy participants (seven females, three males, 27 ± 6 years, 170 ± 8 cm, 69 ± 16 kg) underwent a 4‐week supervised, unilateral knee extensor force accuracy training intervention. The coefficient of variation for force (FORCE^CoV^) and sinusoidal wave force tracking accuracy (FORCE^Sinu^) were determined at 25% maximal voluntary contraction (MVC) pre‐ and post‐training. Intramuscular electromyography was utilised to record individual MU potentials from the vastus lateralis (VL) muscles at 25% MVC during sustained contractions, pre‐ and post‐training. Knee extensor muscle strength remained unchanged following training, with no improvements in unilateral leg‐balance. FORCE^CoV^ and FORCE^Sinu^ significantly improved in only the trained knee extensors by ∼13% (*P* = 0.01) and ∼30% (*P* < 0.0001), respectively. MU FR variability significantly reduced in the trained VL by ∼16% (*n* = 8; *P* = 0.001), with no further alterations to MU FR or neuromuscular junction transmission instability. Our results suggest muscle force control and tracking accuracy is a trainable characteristic in the knee extensors, which is likely explained by the reduction in MU FR variability which was apparent in the trained limb only.

## 1 INTRODUCTION

The human motor unit (MU) is the final component of the neuromuscular system and is fundamental to muscle force generation (Heckman & Enoka, 2012). Each MU comprises a single somatic motor neuron, including its axon, distal axonal branches, neuromuscular junctions (NMJ) and associated innervated skeletal muscle fibres. Motor output is governed by supraspinal commands and spinal reflex pathways which collectively control MU firing rate (FR). Thus, modulation of MU FR contributes to the increase and decrease of muscle force generating capacity.

During muscle contraction, the desired force output fluctuates around an average value rather than being at a constant level (Enoka & Farina, 2021; Pethick & Piasecki, 2022). Variation in MU FR has been identified as a critical determinant influencing the control of muscle force (Enoka & Farina, 2021; Vila‐Cha & Falla, 2016), with associations between muscle force and MU FR dependent on single MU force, the input–output function of motor neurons and the frequency response of the muscle to transform an activation signal into force (Enoka & Farina, 2021). Using computational models allowing manipulation of key MU parameters (MU FR and MU FR variability), increasing index finger force resulted in MU FR variability of the flexor dorsal interosseous reducing exponentially, corresponding with improved simulated force fluctuations (Moritz et al., 2005). These simulated data are consistent with experimental (Laidlaw et al., 2000) and other simulated observations (Enoka et al., 2003) supporting evidence that MU FR variability (i.e., the variability of inter‐discharge intervals across consecutive MU firings) is a, if not the, key physiological parameter influencing the ability to maintain steady muscle contractions (Vila‐Cha & Falla, 2016). Compared to central MU function, much less is known with respect to peripheral MU features (i.e., NMJ transmission instability) and the influence these may have on muscle force control. Peripheral factors such as the release of acetylcholine at the NMJ, sodium/potassium pump activity, or modification to sodium and/or potassium intracellular and/or extracellular concentrations may alter muscle fibre action potential transmission (Allen et al., 2008). Although this alteration may subsequently impact muscle contraction and thus levels of force control, this has not yet been explored in a longitudinal manner.

The coefficient of variation for force (FORCE^CoV^) has been identified as a significant explanatory variable for multiple performance tasks including balance (Zech et al., 2010), walking (Davis, Alenazy et al., 2020), manual dexterity (Keogh et al., 2019; Kornatz et al., 2005), levels of tremor (Kavanagh et al., 2016; Keogh et al., 2019) and the risk of falling in older adults (Carville et al., 2007; Enoka & Farina, 2021). The use of exercise training strategies (e.g., resistance exercise training (RET)) to improve muscle force control is, therefore, of interest for multiple diverse groups of individuals, including athletes, older adults, and those who are clinically vulnerable. It should be noted that findings from such diverging ranges of populations may not be directly comparable; for example, muscle tremor may present differently in varying physiological states (i.e., influence of aged muscle vs. exercise induced fatigue). Although RET is known to improve muscle strength, the effects of such training programmes on muscle force control/accuracy and MU firing properties remain unclear (Elgueta‐Cancino et al., 2022).

While improvements in knee extensor maximal voluntary contraction (MVC) force were observed following 8 weeks RET (∼80% 1‐repetition maximum (1RM)) in young individuals, neither FORCE^CoV^ nor common drive was altered (Beck et al., 2011). Conversely, performing light‐load (30% 1RM) training led to improvements in both knee extensor strength and both knee extensor and elbow flexor muscle force control (Kobayashi et al., 2014), with the greatest RET‐induced improvements in isometric FORCE^CoV^ occurring in the least steady subjects (Tracy & Enoka, 2006). Offering a potential explanation for improvements in FORCE^CoV^ with RET, 4 weeks of isometric strength training which significantly increased muscle strength also increased MU FR (+3 ± 2.5 pps) during the plateau phase of submaximal muscle contractions and decreased in the MU recruitment threshold (Del Vecchio et al., 2019). Similarly, an increase in MU FR during the plateau phase of trapezoidal dorsiflexor contractions was observed following strength training (Kim et al., 2019), with conduction of ballistic muscle contractions leading to earlier activation of MUs and increased MU FR in the dorsiflexor muscles post‐training (Van Cutsem et al., 1998). Despite RET proving to be mostly an effective training mechanism to improve FORCE^CoV^, RET may not be accessible to all individuals due to its higher intensity, which may pose physical limitations for older individuals (Barry & Carson, 2004) and those who are injured or present with disability. Resultingly, alternative training modalities, with a focus on light‐load/task specific training, still need to be established to circumvent these limitations.

The aim of the current study was, therefore, to investigate the effect of a 4‐week low intensity force accuracy training strategy on levels of knee extensor muscle force control/accuracy and any subsequent alterations to central and peripheral MU function in the vastus lateralis (VL) muscle. We hypothesised that muscle FORCE^CoV^ and sinusoidal wave tracking accuracy (FORCE^Sinu^), but not muscle strength, would improve with this training strategy alongside reduced MU FR variability, and would be observed in the trained limb only.

## 2 METHODOLOGY

### 2.1 Ethical approval

This study was approved by the University of Nottingham Faculty of Medicine and Health Sciences Research Ethics Committee (reference number: C16122016) and was conducted in accordance with the *Declaration of Helsinki*, except for registration in a database. Written informed consent was obtained prior to participation in the study.

### 2.2 Participant characteristics

Ten young healthy participants (seven females, three males, 27 ± 6 years, 170 ± 8 cm, 69 ± 16 kg) recruited from the University of Nottingham staff and student population completed this study, each of whom was required to undergo two assessment visits (pre‐ and post‐training) separated by 4 weeks of unilateral (right leg) knee extensor force accuracy training. Participants arrived at the laboratory for assessment visits at ∼09.00 h (±1 h) having undergone an overnight fast (>10 h), with post‐training testing occurring ∼48 h after the last training session. All experimental techniques were completed bilaterally on assessment days. Participants were recreationally active but not actively competing in any sport or formal exercise training regime and were excluded from the study if they presented with any musculoskeletal injury or neurological conditions. As we have previously demonstrated no sex differences in the MU parameters assessed herein (Guo et al., 2022), both sexes were included in the current study.

### 2.3 Assessment of muscle strength, FORCE^CoV^, FORCE^Sinu^ and unilateral balance

Knee extensor muscle strength was assessed via MVCs. Participants sat in a purpose‐built dynamometer with hips and knees flexed at ∼90° and each leg secured via a non‐compliant strap to a force transducer just above the malleoli. After a standardised warm‐up (10 sustained contractions held for 5–10 s, at ∼50% MVC), participants were instructed to perform an MVC. Three attempts were made, each separated by 60 s, with real‐time visual feedback (using Spike2 software v9.06; Cambridge Electronic Design, Cambridge, UK) and strong verbal encouragement provided. The highest peak force (N) was determined as maximal.

**FIGURE 1:**
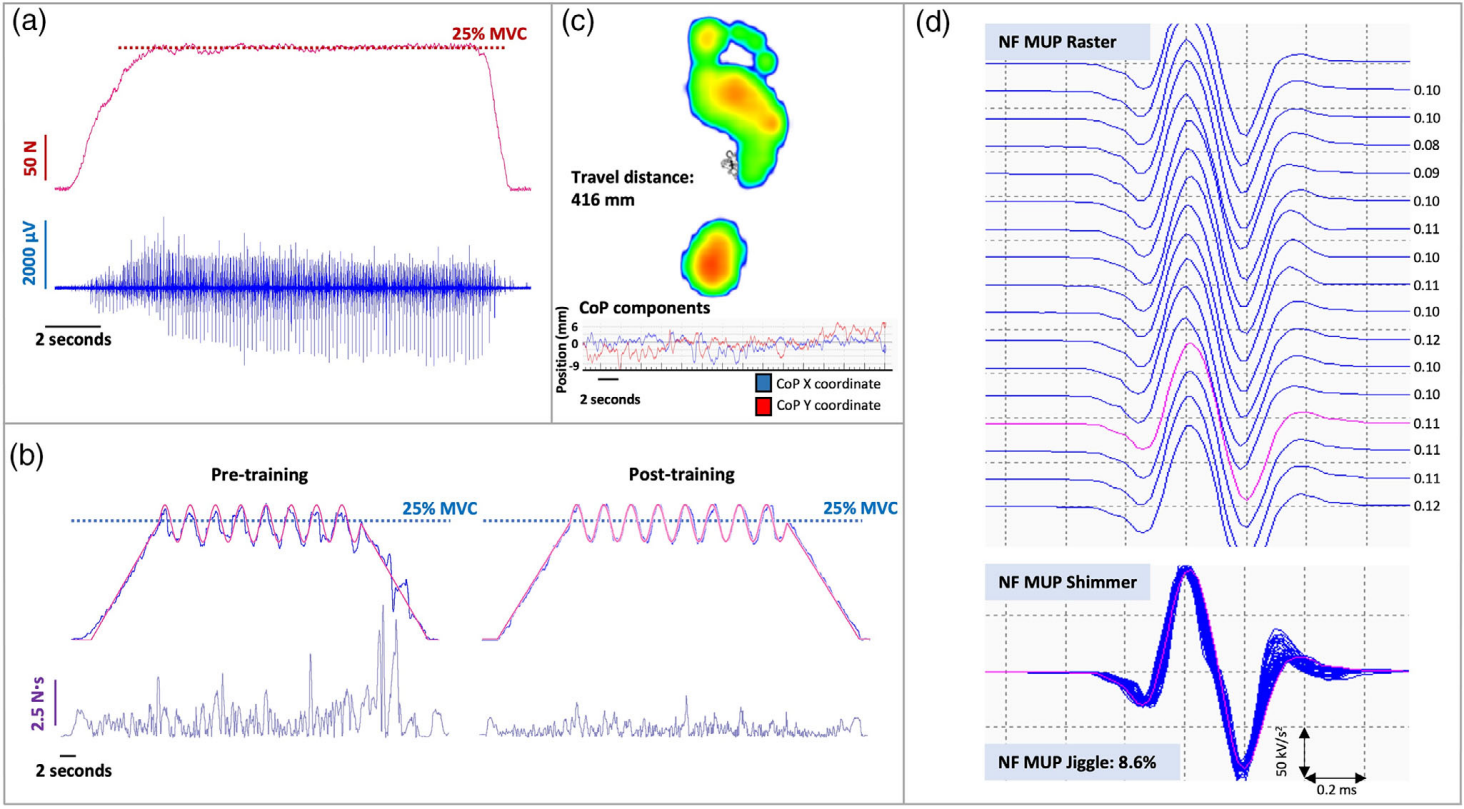
(a) Example force and intramuscular electromyography (iEMG) data recorded during a sustained isometric muscle contraction at 25% maximal voluntary contraction (MVC) in the vastus lateralis muscle. (b) Example raw data from a sinusoidal force tracking task at 25% MVC pre‐ and post‐training in the trained limb. The red line represents the requested target force, whilst the blue line represents the observed force. Subsequent calculation of area under the curve (N s) allowed for quantification of force tracking accuracy following training. (c) Unilateral balance data allowing measurement of centre of pressure (CoP), and the displacement of this (travel distance, mm), during static one‐legged standing. (d) Example raster and shimmer plots of near fibre motor unit potentials (NF MUP) extracted from decomposed iEMG recordings from the vastus lateralis muscle during 25% MVC, allowing quantification of NF MUP jiggle, an indicator of neuromuscular junction transmission instability. Inter‐discharge interval timings (ms) are indicated for each NF MUP firing in the motor unit potential train

Knee extensor FORCE^CoV^ was quantified from a set target line of 25% MVC (Figure 1a). Participants were given a single familiarisation trial before performing six contractions at this intensity. Each contraction lasted 12–15 s with a rest of 30 s between contractions. To avoid corrective actions when reaching the target line, the first two passes were excluded from the calculation. From these six contractions, the mean FORCE^CoV^ was subsequently calculated as (standard deviation/mean) × 100, from the plateau phase of the contraction.

Participants next completed a series of sinusoidal wave force tracking tasks (using OTBioLab software, OT Bioelettronica, Turin, Italy) at 25% MVC to assess levels of FORCE^Sinu^. A familiarisation contraction was performed prior to the assessment contraction. Contractions consisted of eight oscillations at a set amplitude (±4%) lasting for 30 s. A 10‐s ramp preceded and followed each oscillating section of the contraction to allow force to steadily increase and decrease to and from the desired contraction intensity (Figure 1b). Contractions were exported and analysed in Spike2 (version 9) software, where a virtual channel was created (by subtracting the performed path from the requested path, and rectifying) and the area under the curve (N s) of this channel was representative of the level of deviation from the target line, reflecting muscle force tracking accuracy.

Participants also completed physical function tests to assess unilateral balance of both legs pre‐ and post‐training. All balance tests were performed using a Footscan plate (Footscan, 200 Hz, RSscan International, Paal, Belgium) allowing measurement of centre of pressure, and the displacement of this, during static one‐legged standing. Participants were asked to visually focus on a fixed point in front of them for the duration of the test (30 s). A 5‐s countdown was given before instruction to lift one leg, 2 s before the recording period began. Distance travelled (mm), the displacement of centre of pressure, was recorded for further analysis (Figure 1c).

### 2.4 Intramuscular EMG measures

#### 2.4.1 Motor point identification

The motor point of the VL was identified as the site of the muscle that produced the largest localised visible twitch using a low stimulation current with a cathode probe (Medserve, Daventry, UK) and a self‐adhesive anode electrode (Dermatode, Fermadomo, BR Nuland, the Netherlands) (Piasecki, Ireland, Coulson et al., 2016). A constant current stimulator (Digitimer DS7AH, Digitimer, Welwyn Garden City, UK) was set to a compliance voltage of 400 V with a 50 μs pulse width.

#### 2.4.2 Sampling of single motor units during voluntary contractions

Intramuscular electromyography (iEMG) recordings were obtained using disposable concentric needle electrodes with a recording area of 0.07 mm^2^ (model N53153, Teca, Hawthorne, NY, USA), with a grounding electrode on the patella. Participants were asked to relax their muscles to enable insertion of the needle electrode into the VL muscle to enable sampling of MUs during the series of voluntary isometric contractions used to assess FORCE^CoV^ (as described above). Following each contraction, the needle electrode was withdrawn 5–10 mm and the bevel rotated 180°, recording from a total of four to six contractions from spatially distinct areas (Jones et al., 2021). iEMG signals were sampled at 50 kHz and bandpass filtered at 10 Hz to 10 kHz. Signals were digitised with a CED Micro 1401 data acquisition unit (Cambridge Electronic Design). All iEMG and force signals were recorded and displayed in real‐time via Spike2 software (version 9). Data were analysed offline in Spike2 (v9.06).

#### 2.4.3 iEMG signal analysis

iEMG data are available for eight participants, as too few motor unit potentials (MUPs; <3) were isolated in the control limb post‐intervention in two participants. Procedures for identifying, recording and analysing individual MUPs and calculating near fibre (NF) parameters have been described in detail elsewhere (Piasecki, Ireland, Stashuk et al., 2016; Stashuk, 1999). Decomposition‐based quantitative electromyography (DQEMG) software was used for all iEMG signal analysis and visual inspection of all MUPs and NF‐MUPs was performed. Sets of individual MUPs generated by a single MU were identified in the iEMG signal and extracted into MUP trains (MUPT). MUPTs composed of MUPs generated by more than one MU or having fewer than 40 MUPs were excluded.

Individual MUPs within a MUPT were further used to assess MU FR and NF jiggle. MU FR is expressed in Hz. MU FR variability was calculated as the coefficient of variation of the MU inter‐discharge‐interval times and expressed as a percentage. A NF MUP (Figure 1d) was obtained by calculating the slope of its corresponding MUP, using a low‐pass second‐order differentiator (Piasecki, Garnés‐Camarena et al., 2021). This effectively reduces the uptake area of the needle electrode, thus ensuring only near fibres significantly contribute to a detected NF MUP. NMJ transmission instability is quantified as NF jiggle, a measure of the variability of consecutive NF MUP shapes across a train (Piasecki, Garnés‐Camarena et al., 2021; Stålberg & Sonoo, 1994). A total of 1271 MUPs were recorded from all participants across all contractions from both legs and time points (pre‐ and post‐training). This consisted of a total of 649 in the trained limb and 622 in the untrained limb. A mean of 8 ± 3 MUPs were sampled with each needle position.

### 2.5 Force accuracy training

All participants were required to perform force accuracy training 3×/week for 4 weeks, with all sessions being fully supervised by a member of the research team. Training was completed unilaterally for the knee extensors, with all participants training the right leg, which was also the dominant limb for all volunteers. Participants completed six sinusoidal force tracking contractions (as described previously and illustrated in Figure 1b) for each training session, with contractions conducted at 10% MVC (x2), 25% MVC (x2) and 40% MVC (x2), determined in the pre‐training assessment visit, in a randomised order. The number of oscillations (6, 8 or 10) and amplitude (±2%, ±4% or ±8%) of the sinusoidal waves was different from that used in experimental testing (as stated above) and was again randomised for each training session. Contractions lasted 20–30 s dependent on contraction intensity and were separated by 60 s rest.

### 2.6 Statistical analysis

Data are presented as mean ± SD unless stated otherwise. As there were no bilateral leg differences at baseline in any parameter, muscle strength (i.e., MVC force), FORCE^CoV^, FORCE^Sinu^ and distance travelled during unilateral leg balance were analysed using a two‐way repeated measures ANOVA (condition × time) to assess measures in both the trained and untrained legs pre‐ and post‐training. Bonferroni *post hoc* tests were used to identify statistical differences as a result of training. Multi‐level mixed effects linear regression models were used to analyse MU data at 25% MVC with leg (trained vs. untrained) and time (pre vs. post) as factors. Interactions were first examined, and where present, individual coefficients for each leg are reported. Adjusted beta values and 95% confidence intervals (CIs) are reported for all parameters. Statistical significance was accepted at *P* < 0.05. Data analysis was performed using GraphPad Prism Version 8 (GraphPad Software, San Diego, CA, USA) for analysis involving two‐way repeated‐measures ANOVA, and Stata SE version 16 (StataCorp, College Station, TX, USA) for multi‐level mixed effects regression.

## 3 RESULTS

### 3.1 Muscle strength and unilateral balance

**FIGURE 2:**
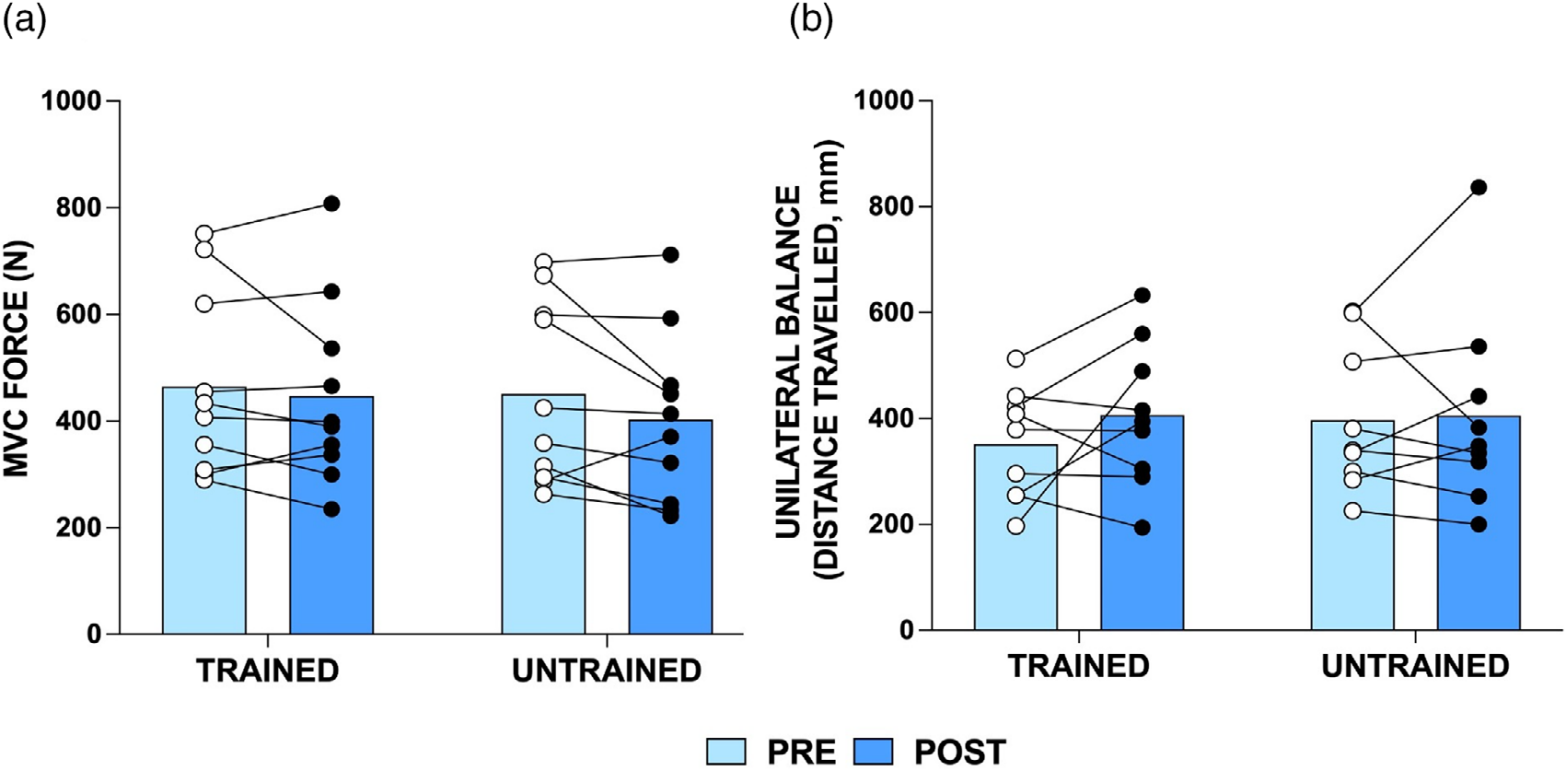
Knee extensor maximum voluntary contraction (MVC) force (N) (a) and displacement of centre of pressure during unilateral balance tasks (b) in *n* = 10 young individuals (*n* = 9 for unilateral balance due to one participant not achieving the minimum balance time required time to complete the test) pre‐ and post‐training in the trained and untrained limb. Group means shown as bars with individual data overlaid

Knee extensor maximal isometric strength remained unchanged following 4 weeks’ unilateral force accuracy training (Figure 2a; condition × time interaction effect: *P* = 0.39; main effect of time: *P* = 0.08) in both the trained (464.4 ± 173.4 vs. 447.0 ± 173.1 N, *P* = 0.97) and untrained (450.6 ± 171.6 vs. 403.0 ± 160.7 N, *P* = 0.13) legs.

Displacement of the centre of pressure during unilateral static standing did not change (condition × time interaction effect: *P* = 0.45; main effect of time: *P* = 0.30), and thus no improvements in unilateral balance were observed in either leg following force accuracy training (Figure 2b; *n* = 9; trained: 352.0 ± 105.4 vs. 406.6 ± 137.8 mm, *P* = 0.42; untrained: 397.2 ± 138.6 vs. 405.9 ± 189.2 mm, *P* > 0.99).

### 3.2 Muscle force tracking accuracy

**FIGURE 3:**
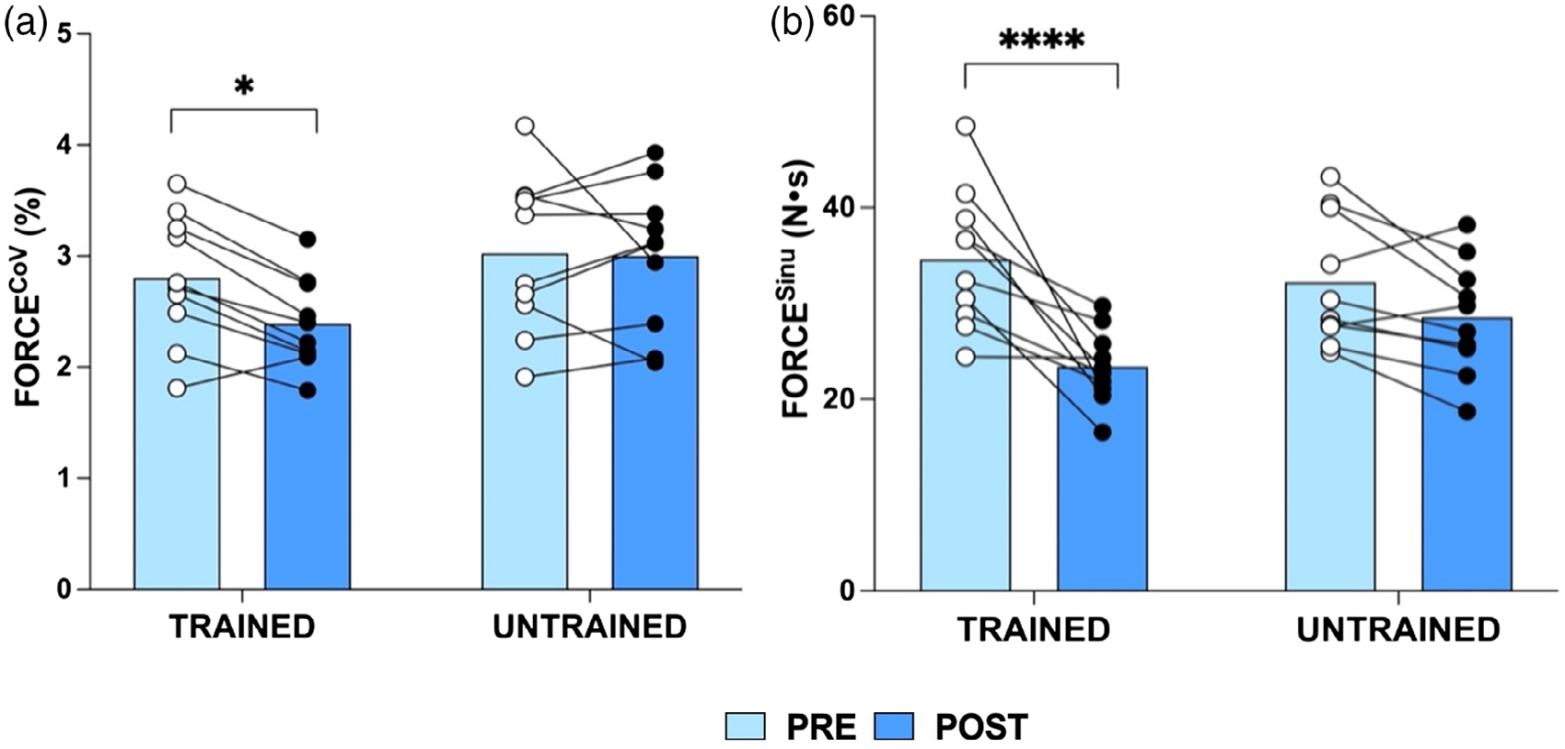
Knee extensor coefficient of variation for force (FORCE^CoV^, %) (a) and knee extensor sinusoidal wave force tracking accuracy (FORCE^Sinu^; calculated as area under the curve; N s) (b) at 25% maximal voluntary contraction pre‐ and post‐training in the trained and untrained legs of *n* = 10 young individuals. Group means shown as bars with individual data overlaid. *P < 0.05, ****P < 0.0001

Although the interaction effect was not significant (*P* = 0.053), there was a main effect of time (*P* = 0.03), with the trained knee extensors demonstrating a significant improvement in FORCE^CoV^ following force accuracy training (trained: 2.80 ± 0.58% vs. 2.39 ± 0.40%, *P* = 0.01; untrained: 3.02 ± 0.71% vs. 3.00 ± 0.65%, *P* > 0.99; Figure 3a).

A significant interaction effect (condition × time, *P* = 0.02) and main effect of time (*P* < 0.0001) was observed for knee extensor FORCE^Sinu^ (Figure 3b), with improvements in FORCE^Sinu^ in the trained (34.59 ± 7.26 vs. 23.40 ± 3.85 N s, *P* < 0.0001) but not the untrained (32.22 ± 6.76 vs. 28.57 ± 5.93 N s, *P* = 0.19) leg following force accuracy training.

When including the eight individuals with complete iEMG data at all time points, the same pattern was true for FORCE^CoV^ (condition × time, *P* = 0.012, trained leg: *P* = 0.012, untrained leg: *P* = 0.86) and FORCE^Sinu^ (condition × time, *P* = 0.032, main effect of time, *P =* 0.001, trained leg: *P =* 0.0009, untrained leg: *P* = 0.496).

### 3.3 Motor unit features

**FIGURE 4:**
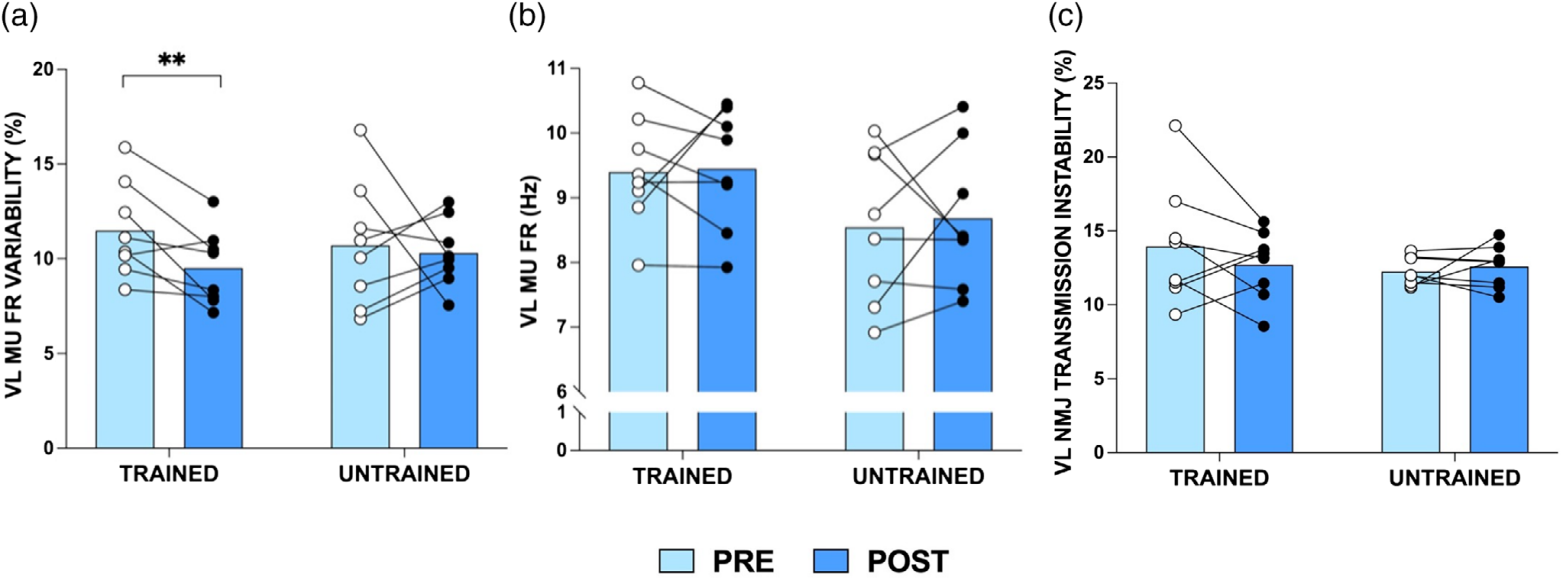
Vastus lateralis (VL) motor unit (MU) firing rate (FR) variability (%) (a), VL MU FR (Hz) (b), and VL neuromuscular junction transmission (NMJ) instability (%) (c) at 25% maximal voluntary contraction, pre‐ and post‐training (*n* = 8), in the trained and untrained legs. Group means shown as bars with individual data overlaid for data visualisation only. All analyses were based on multi‐level linear regression models, where MUs were clustered to each muscle/participant. **P = 0.001

VL MU FR variability (Figure 4a) displayed a significant interaction effect (*P* = 0.041) following training, with decreased MU FR variability in the trained leg (β = −2.018, 95% CI: −3.202, −0.835; *P* = 0.001) but no change in the untrained leg (β = 0.862, 95% CI: −0.271, 1.995; *P* = 0.14). No changes were observed in VL MU FR (Figure 4b; trained leg: β = 0.011, 95% CI: −0.319, 0.541; *P* = 0.613; untrained leg: β = 0.009, 95% CI: −0.383, 0.401; *P* = 0.97) or NMJ transmission instability in either leg following force accuracy training (Figure 4c; trained leg: β = −0.714, 95% CI: −1.823, 0.399; *P* = 0.208; untrained leg, β = 0.239, 95% CI: −0.856, 1.324; *P* = 0.67).

## 4 DISCUSSION

The aim of the current study was to investigate the effects of a force accuracy training programme, with respect to muscle FORCE^CoV^ and alterations to individual MU features in VL. We highlight that 4 weeks of force accuracy training resulted in improvements in muscle force accuracy and control, evidenced through improved FORCE^CoV^ and FORCE^Sinu^ in the trained knee extensors only. No changes in MVC force or displacement of pressure during unilateral balance tasks were observed with this form of training. The improvements in force accuracy and control were accompanied by decreased MU FR variability, again occurring in the trained limb only. Despite no changes in MVC force (i.e., muscle strength), low‐intensity force accuracy training led to improvements of force control/accuracy and associated improvements in MU parameters. This form of low‐intensity intervention may be particularly relevant to older adults, clinically vulnerable individuals and/or hospitalised patients.

In line with our hypothesis, MVC force remained unchanged in the knee extensors across both legs. However, these results are not in direct agreement with others. For example, in older adults, low intensity RET (30% 1RM) increased knee extensor MVC after both 8(Kobayashi et al., 2014) and 16(Tracy & Enoka, 2006) weeks of training, with numerous studies reporting that low intensity RET (i.e., ∼20–40% 1RM) can induce increases in muscle strength (Hortobagyi et al., 2001; Keen et al., 1994; Kobayashi et al., 2014; Tracy & Enoka, 2006). This notion does, however, remain inconclusive with studies such as that by Moore et al. (2004) reporting unchanged isometric elbow flexor strength following 8 weeks’ RET in young individuals at 50% 1RM. Considering the current study, we suggest an absence of increased strength following our force accuracy training may have occurred due to: (i) the relatively low forces applied during the intervention, (ii) a fixed training load (i.e., %MVC was not increased throughout training), and (iii) studying a population of healthy young individuals, with previous studies of similar methodology often recruiting previously sedentary older adults, a population arguably having the most to gain from being physically active (McPhee et al., 2016).

We demonstrate no functional improvements in the displacement of centre of pressure during unilateral balance tasks post‐training, suggesting FORCE^CoV^ of the knee extensors did not influence unilateral balance. Previous studies of other muscles have positively associated FORCE^CoV^ with balance measures (Davis, Allen et al., 2020; Kouzaki & Shinohara, 2010; Oshita & Yano, 2010). For example, FORCE^CoV^ assessed via low intensity isometric contractions in the plantar flexors was significantly associated with balance performance with eyes closed (determined by the time, in seconds, to complete the test) in both young and older individuals. However, one study demonstrated this association for contractions at 20%, but not 10% MVC (Oshita & Yano, 2010), and another for contraction intensities ≤5% MVC (determined via displacement of centre of pressure) (Kouzaki & Shinohara, 2010). FORCE^CoV^ of the hip abductors and dorsiflexors has been shown to be the most significant explanatory variable in sway‐area rate during light load contractions, although most of the variance across conditions was unexplained,suggesting other physiological mechanisms important for postural control likely influence unilateral balance (Davis, Allen et al., 2020). The effects of force accuracy training on functional outcomes such as unilateral balance remains, therefore, to be further examined in other muscle groups and populations such as older individuals, who display deterioration of unilateral balance with advancing age (Maki et al., 1990; Izquierdo et al., 1999; Hess & Woollacott, 2005).

The current study utilised two different force tracking tasks, varying in difficulty, to assess levels of FORCE^CoV^ and force tracking accuracy. Our results demonstrate improvements in both FORCE^CoV^ and FORCE^Sinu^ following training. During isometric contractions of fluctuating force (i.e., sinusoidal contractions), the recruitment and subsequent de‐recruitment of MUs needs to be aligned to match the desired trajectory to increase and decrease force (Duchateau & Enoka, 2008) and as such, they require different control strategies. Resultingly, the fluctuation in force during the sinusoidal contractions may contribute to positive alterations of force tracking and control strategies.

Commonly, FORCE^CoV^ has been highlighted as a critical explanatory variable with respect to muscular performance of tasks including walking (Davis, Alenazy et al., 2020), tremor (Kavanagh et al., 2016; Keogh et al., 2019) and risk of falls in older adults (Carville et al., 2007). FORCE^CoV^ is also associated with impaired functional ability in multiple sclerosis (Davis, Alenazy et al., 2020), and progressively deteriorates from middle to older age in highly active males and females (Piasecki, Inns et al., 2021). Therefore, the impact of force accuracy training on FORCE^CoV^ should be examined in older/clinically vulnerable populations to determine whether the more targeted approach (vs. traditional RET, for example) leads to improvements in force accuracy and control and consequently may aid with performance of functional tasks.

No alterations to MU FR were noted in the present study, which may be due to the low intensity of training, as 4 weeks of strength training at ∼75% MVC increased tibialis anterior MU FR during the plateau phase of submaximal isometric contractions (at 35, 50 and 70% MVC) and reduced MU recruitment thresholds (Del Vecchio et al., 2019). Similar effects on MU FR were also reported in the abductor digiti minimi when training was performed at a maximal intensity (Patten et al., 2001). Conversely, another study demonstrated increased VL MU FR at 100% MVC but not during submaximal contractions following 6 weeks of training using dynamic muscle contractions (Kamen & Knight, 2004), whilst 2 weeks of force modulation training (i.e., neuromuscular control training) reduced MU FR during contractions at 30–60% MVC (Patten & Kamen, 2000). Taken together, it is likely that adaptation to MU FR is muscle and contraction level specific, and sensitive to the form of training intervention (Elgueta‐Cancino et al., 2022).

In line with our hypothesis and the work of others (Laidlaw et al., 2000; Enoka et al., 2003; Moritz et al., 2005; Enoka & Farina, 2021), although not unanimous (Beck et al., 2011), we demonstrate a significant reduction in MU FR variability following force accuracy training. In flexor dorsal interosseous, Kornatz and colleagues highlight a weak association between reduced force fluctuations, in addition to improvements in manual dexterity, and declines in MU FR variability (Kornatz et al., 2005), which was later strengthened using computational models (Moritz et al., 2005). Furthermore, MU FR variability was reported to reduce, as such enhancing knee extensor FORCE^CoV^, following strength but not endurance training, suggesting different training modalities result in varying neuromuscular adaptations (Vila‐Cha & Falla, 2016). MU FR variability and force fluctuations are governed by central descending pathways modulated by independent and common synaptic inputs to the MU pool (Taylor et al., 2003; Vila‐Cha & Falla, 2016), and here we demonstrate improvements in force accuracy and MU FR variability in the trained limb only, which may be a result of reduced antagonist muscle activity and/or inhibitory afferent feedback (Enoka & Farina, 2021). Further, common inputs of descending and sensory signals induce a correlation between low‐frequency oscillations in FR of motor neurons, known as common drive, also significantly influence fluctuations in muscle force output (Negro et al., 2009). Interestingly, low‐frequency oscillatory components of MU FR were strongly associated to explain most variation in muscle force output during submaximal contractions, with MU FR variability being poorly correlated with FORCE^CoV^ (Negro et al., 2009). Therefore, although not assessed in the current study, the influence and potential alterations to common drive should additionally be considered as a mechanism to aid improvements in FORCE^CoV^. There is potential for ionic changes (e.g., modification to sodium and/or potassium ion intracellular and/or extracellular concentrations, subsequently altering muscle fibre action potential transmission; Allen et al., 2008), release of acetylcholine at the NMJ, and/or the type/intensity of muscle contraction (Enoka et al., 2003; Carville et al., 2007; Enoka & Farina, 2021) to also influence levels of muscle force control; however, we observed no alterations to NMJ transmission instability, assessed via NF MUP jiggle.

### 4.1 Strengths and limitations

As the training period was only 4 weeks, it offers translational relevance and application to pre/rehabilitation scenarios (Durrand et al., 2019) with, for example, pre‐operative colorectal patients in the UK having a 31‐day target time frame between decision to treat and operation (Boereboom et al., 2019). The short duration of each training session (∼20 min) also counters one of the most commonly cited barriers to exercise interventions: ‘lack of time’ (Trost et al., 2002). Secondly, the tasks constituting force accuracy training are arguably more applicable to daily movements (e.g., rising from a chair) than traditional RET due to the fluctuations in force requiring greater muscle coordination. The use of iEMG and near fibre analysis to identify single MU features from a range of muscle depths in both male and female participants affords greater insight into central and peripheral adaptions at a single MU level than commonly achieved with surface electromyography methodology. A limitation of the study is that all MUs were sampled at 25% MVC which reveals little of adaptations that may be occurring with higher threshold MUs. Furthermore, despite providing great detail, an inherent limitation of iEMG is the inability to reliably track the same MUs pre‐ and post‐intervention (Martinez‐Valdes et al., 2017). Finally, reduced antagonist co‐activation cannot be ruled out in the observed improvements in force tracking accuracy (De Luca & Mambrito, 1987).

### 4.2 Conclusion

To summarise, we highlight that a 4‐week period of targeted force accuracy leads to improved muscle force control and accuracy in young healthy participants, which is associated with reduced MU FR variability. Importantly, these adaptations and possible mechanisms were evident in the trained limb only. These findings may influence interventional strategies to improve force accuracy, including in older and clinical populations where such improvements may help with independence maintenance via improved performance of activities of daily living.

## COMPETING INTERESTS

The authors declare no competing interests.

## AUTHOR CONTRIBUTIONS

All authors contributed to the conception and design of the work. Isabel A. Ely, Eleanor J. Jones, Thomas B. Inns, Síobhra Dooley and Sarah B. J. Miller acquired the data and Isabel A. Ely analysed the data. Isabel A. Ely, Daniel W. Stashuk, Philip J. Atherton, Bethan E. Phillips and Mathew Piasecki drafted the manuscript and prepared the figures. All authors approved the final version of the manuscript. All authors agree to be accountable for all aspects of the work in ensuring that questions related to the accuracy or integrity of any part of the work are appropriately investigated and resolved. All persons designated as authors qualify for authorship, and all those who qualify for authorship are listed.

## FUNDING INFORMATION

This work was supported by the Medical Research Council (MR/P021220/1) as part of the MRC‐Versus Arthritis Centre for Musculoskeletal Ageing Research awarded to the Universities of Nottingham and Birmingham and by the NIHR Nottingham Biomedical Research Centre.

## Supporting information

Statistical Summary Document

## Data Availability

The datasets generated and analysed during the current study are available from the corresponding author upon reasonable request.
