# Supplementary material for: Training‐induced improvements in knee extensor force accuracy are associated with reduced vastus lateralis motor unit firing variability
Source: Exp Physiol. 2022 Aug 12;107(9):1061–70. doi: 10.1113/EP090367 (PMC9542263; doi:10.1113/EP090367)
Supplement: Supplementary file 1 — Statistical Summary Document [file EPH-107-1061-s001.docx]

**Manuscript Title: Training induced improvements in knee extensor force accuracy are associated with reduced vastus lateralis motor unit firing variability**

**Authors:** Isabel A Ely, Eleanor J Jones, Thomas B Inns, Síobhra Dooley, Sarah B J Miller, Daniel W Stashuk, Philip J Atherton, Bethan E Phillips, Mathew Piasecki

**Animal model used, if applicable:** N/A

**Underlying hypothesis:** We hypothesised that the coefficient of variation for force (FORCE^CoV^) and sinusoidal wave tracking accuracy (FORCE^Sinu^) would improve following force accuracy training strategy, alongside reduced MU FR variability, and would be observed in the trained limb only.

**Definitions of ‘n’:**

Question 1: n = number of participants for measures of knee extensor muscle strength pre- and post-force accuracy training

Question 2: n = number of participants for measures of unilateral balance pre- and post-force accuracy training

Question 3: n = number of participants for measures of knee extensor force accuracy (both FORCE^CoV^ and FORCE^Sinu^) pre- and post-force accuracy training

Question 4: n = number of participants for vastus lateralis (VL) MU parameters (MU firing rate (FR), MU FR variability and neuromuscular junction (NMJ) transmission instability) pre- and post-force accuracy training.

**Statistical summary table:**

| Experimental question number* | Finding/ conclusion | Experimental location/ variable  e.g. muscle, neocortex or genotype | Mean value  (or other summary statistic) | SD | n val. | P** | Units | Data comparisons  e.g. WT vs KO | Statistical test | Any other variable  e.g. subjects’ age or sex | Figure/ table in which data are presented | Comments  e.g. observation |
| --- | --- | --- | --- | --- | --- | --- | --- | --- | --- | --- | --- | --- |
| 1 | No change in muscle strength in either limb post-training | Knee extensor maximal strength (TRAINED LIMB) | PRE: 464.4; POST: 447.0 | PRE: 173.4; POST: 173.1 | 10 | 0.97 | N | Pre vs post | 2-way ANOVA  Bonferroni multiple comparisons |  | 2A | Condition*time: *p*=0.39; main effect of time: *p*=0.08 |
|  |  | Knee extensor maximal strength (UNTRAINED LIMB) | PRE: 450.6; POST: 403.0 | PRE: 171.6; POST: 160.7 | 10 | 0.13 | N | Pre vs post |  |  |  |  |
| 2 | No change in unilateral balance in either limb post-training | Displacement of pressure (TRAINED LIMB) | PRE: 352.0; POST: 406.6 | PRE: 105.4; POST: 137.8 | 9 | 0.42 | mm | Pre vs post | 2-way ANOVA  Bonferroni multiple comparisons |  | 2B | condition*time: *p*=0.45; main effect of time: p=0.30 |
|  |  | Displacement of pressure (UNTRAINED LIMB) | PRE: 397.2; POST: 405.9 | PRE: 138.6; POST: 189.2 | 9 | >0.99 | mm | Pre vs post |  |  |  |  |
| 3 | Improvements in FORCE^CoV^ for the trained limb only | Knee extensor FORCE^CoV^ (TRAINED LIMB) | PRE: 2.80; POST: 2.39 | PRE: 0.58; POST: 0.40 | 10 | **0.01** | % | Pre vs post | 2-way ANOVA  Bonferroni multiple comparisons |  | 3A | condition*time: *p*=0.053; main effect of time: **p=0.03** |
|  |  | Knee extensor FORCE^CoV^ (UNTRAINED LIMB) | PRE: 3.02; POST: 3.00 | PRE: 0.71; POST: 0.65 | 10 | >0.99 | % | Pre vs post |  |  |  |  |
|  | Improvements in FORCE^Sinu^ for the trained limb only | Knee extensor FORCE^Sinu^ (TRAINED LIMB) | PRE: 34.59; POST: 23.40 | PRE: 7.26; POST: 3.85 | 10 | **<0.0001** | N**·**s | Pre vs post | 2-way ANOVA  Bonferroni multiple comparisons |  | 3B | condition*time: ***p*=0.02**; main effect of time: ***p*<0.0001** |
|  |  | Knee extensor FORCE^Sinu^ (UNTRAINED LIMB) | PRE: 32.22; POST: 28.57 | PRE: 6.76; POST: 5.93 | 10 | 0.19 | N**·**s | Pre vs post |  |  |  |  |
| 4 | MU FR variability reduced in the trained limb only with no other changes to other MU parameters assessed | VL MU FR variability (TRAINED LIMB) | ß=-2.018 | 95% CI=[-3.202]-[-0.835] | 8 | **0.001** | % | Pre vs post | Multi-level mixed effects linear regression models**^+^** | **^+^** MUs pooled to control for differences between subjects, timepoints and treatment conditions (i.e., looking pre vs post in both limbs but controlling for subjects as to not artificially increase the N and to avoid pseudo-replication) | 4A | Interaction effect: ***p*=0.041** |
|  |  | VL MU FR variability (UNTRAINED LIMB) | ß=0.862 | 95% CI=[-0.271]-[1.995], | 8 | 0.14 | % | Pre vs post |  |  |  |  |
|  |  | VL MU FR (TRAINED LIMB) | ß=0.011 | 95% CI=[-0.319]-[0.541] | 8 | 0.613 | Hz | Pre vs post |  |  | 4B |  |
|  |  | VL MU FR (UNTRAINED LIMB) | ß=0.009 | 95% CI=[-0.383]-[0.401] | 8 | 0.97 | Hz | Pre vs post |  |  |  |  |
|  |  | VL NMJ transmission instability (TRAINED LIMB) | ß=-0.714 | 95% CI=[-1.823]-[0.399] | 8 | 0.208 | % | Pre vs post |  |  | 4C | Figure 4 for visualisation only with stats based on multi-level mixed effects linear regression models |
|  |  | VL NMJ transmission instability (UNTRAINED LIMB) | ß=0.239 | 95% CI=[-0.856]-[1.324] | 8 | 0.67 | % | Pre vs post |  |  |  |  |

*You may use multiple lines for the same question to indicate multiple comparisons

** Authors may wish to make the text bold where p is considered significant against a stated confidence limit.
